# Neonatal herpes simplex virus infection combined with neonatal lupus erythematosus: a case reported

**DOI:** 10.3389/fped.2025.1592459

**Published:** 2025-06-12

**Authors:** Haiyan Jiang, Hong Qiu, Minghuan Wang, Haiyin Yang, Li Li, Yanhong Li

**Affiliations:** Neonatal Intensive Care Unit, Women and Children’s Hospital of Ningbo University, Ningbo, Zhejiang, China

**Keywords:** neonatal, herpes simplex virus, neonatal lupus erythematosus, atrioventricular block, case report

## Abstract

**Background:**

Neonatal herpes simplex virus (HSV) infection complicated by neonatal lupus erythematosus (NLE) is rare and is associated with high mortality and poor neurological outcomes in survivors. Enhancing clinical understanding of this condition is essential to reduce misdiagnosis and missed diagnosis.

**Case presentation:**

A neonate presented with complete atrioventricular block (CAVB) as the initial clinical manifestation, which was successfully resolved following combined therapy with isoproterenol, hydrocortisone, and intravenous immunoglobulin (IVIG). Maternal history of HSV infection during pregnancy, coupled with suspected herpetic skin lesions in the neonate, strongly suggested HSV as the putative etiology. Despite initiation of acyclovir therapy for confirmed HSV infection, the recurrence of cutaneous rash prompted further evaluation, leading to the diagnosis of NLE. Notably, no specific treatment was administered for NLE, as the condition remained clinically quiescent. Follow-up at 7 months of age revealed no neurological abnormalities, no recurrence of CAVB, and gradual resolution of the rash.

**Conclusion:**

The clinical presentations of neonatal HSV infection and NLE can overlap. Early diagnosis and intervention are crucial for successful treatment and improved prognosis.

## Introduction

Herpes simplex virus (HSV) is a neurotropic double-stranded DNA virus that can be classified into two types: type I and type II. The virus primarily infects the skin, mucosa, and neural tissues. It is a common viral disease affecting all ethnic groups worldwide, although neonatal HSV infection is relatively rare (1). Neonatal lupus erythematosus (NLE) is an acquired autoimmune disease caused by the transplacental transfer of abnormal anti-SSA and anti-SSB antibodies from the mother, affecting the skin, heart, blood, nerves, and liver (2). In January 2024, a child presenting with complete atrioventricular block (CAVB) as the initial symptom was admitted to our hospital and diagnosed with neonatal HSV infection complicated by NLE. The diagnosis and treatment of this patient were analyzed in detail to provide reference for the diagnosis and management of neonatal HSV infection and the identification of NLE.

## Case description

The female newborn, only at 5 h of life, was admitted due to a “slow heart rate more than 5 h after birth.” Her mother was healthy, underwent regular prenatal examinations, and had no history of gestational diabetes, hypertension, obvious prenatal signs of infection, or connective tissue disease. The child, G1P1, was born at a gestational age of 36 weeks and 6 days via cesarean section due to intrauterine distress. The amniotic fluid, umbilical cord, and placenta were normal, with no premature rupture of membranes. The Apgar score was 10 at both 1 min and 5 min, and the birth weight was 2,700 g. After birth, the baby returned to the ward with her mother, where it was noted that her heart rate was approximately 70 beats/min while at rest, and her body temperature was low. She was kept warm in an incubator and her body temperature reached 36.2℃, but her heart rate was still low. Since there was no neonatal intensive care unit in the local area, she was transferred to our hospital.

Physical examination on admission: The patient's temperature was 34.7°C, heart rate was 86 beats per minute, blood pressure was 61/42 mmHg, TcSO₂96%. He exhibited poor mental responsiveness, pallor, shortness of breath, scattered petechiae, and cyanosis on the face, perioral area, auricles, and buttocks (Figure 4a). The anterior fontanel was flat and soft, the heartbeat was irregular, muscle tone in the limbs was low, and the extremities were cold.

**Figure 1:**
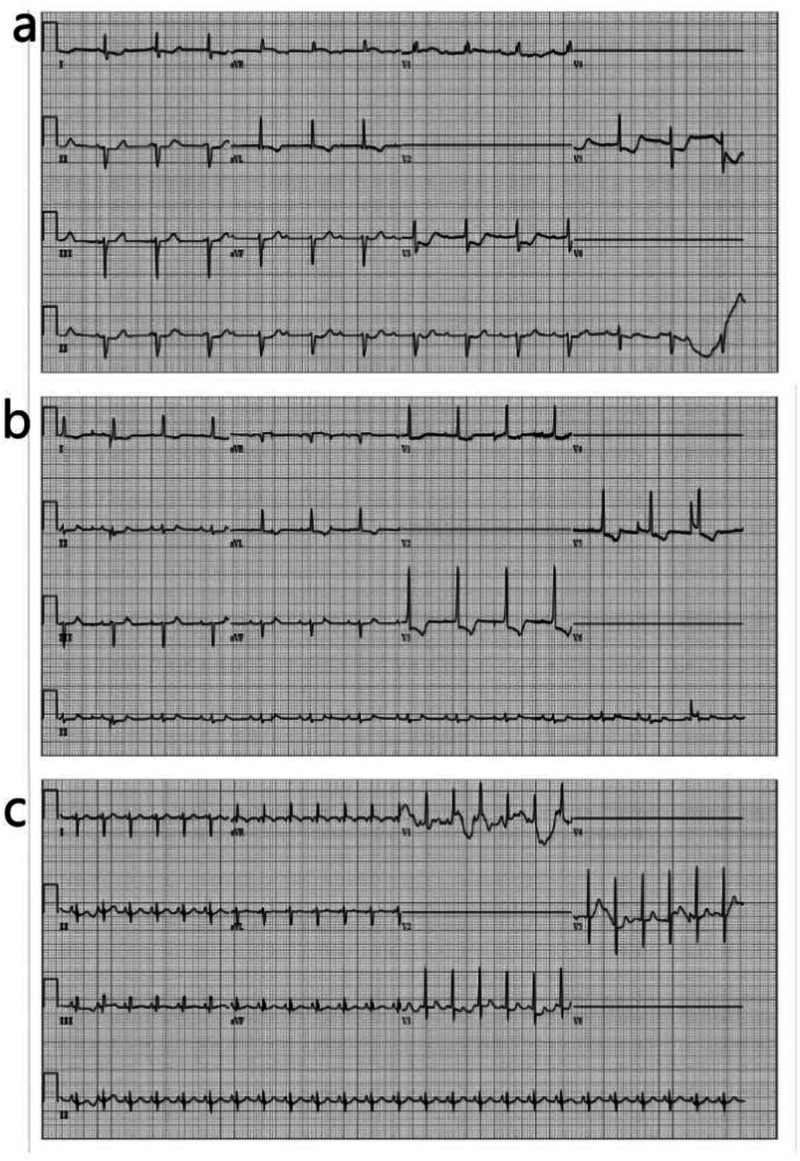
ECG tracing. **(a)** 6 h after birth. **(b)** 16 h after birth. **(c)** 22 h after birth.

**Figure 2:**
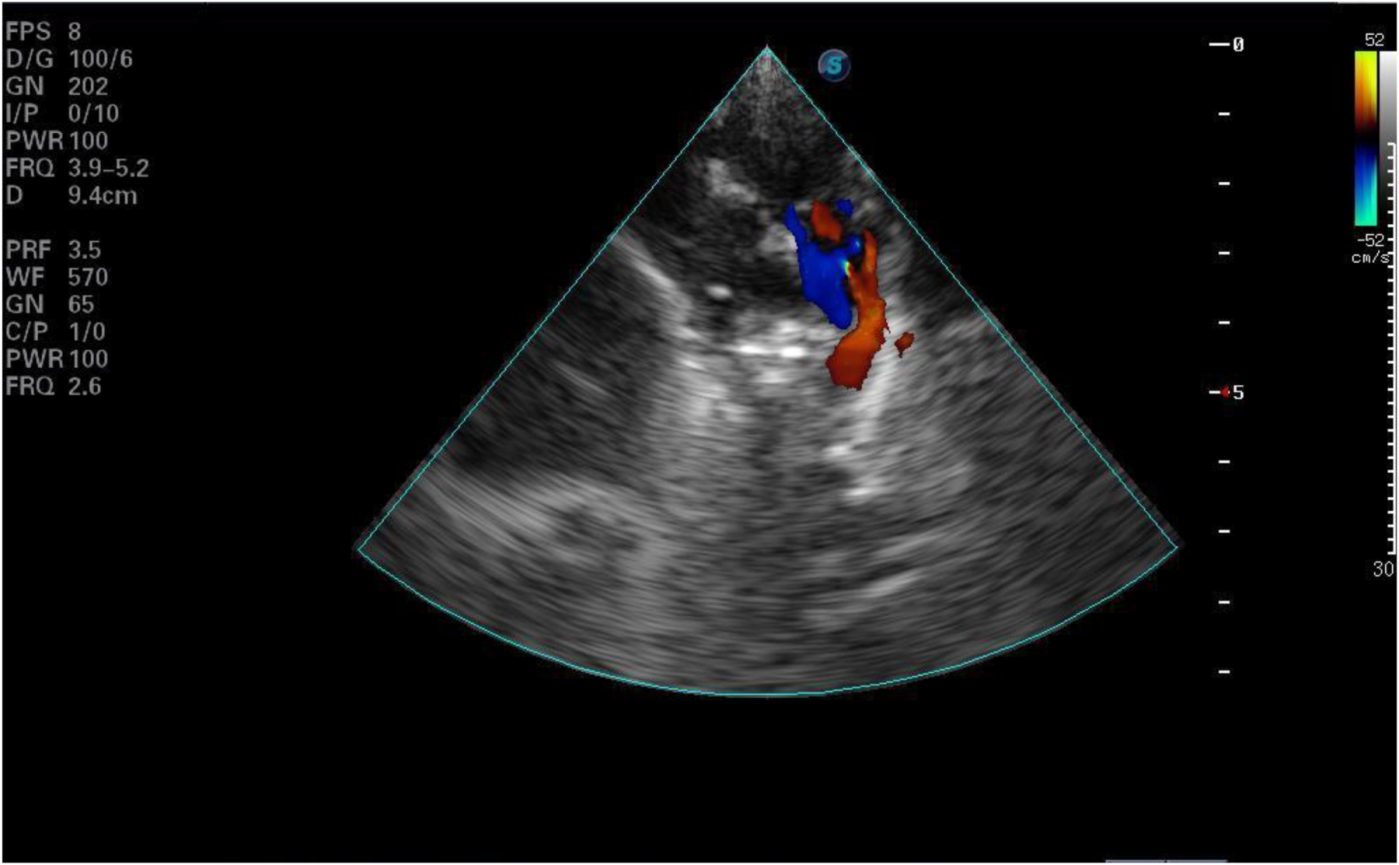
Ventricular septal defect.

**Figure 3:**
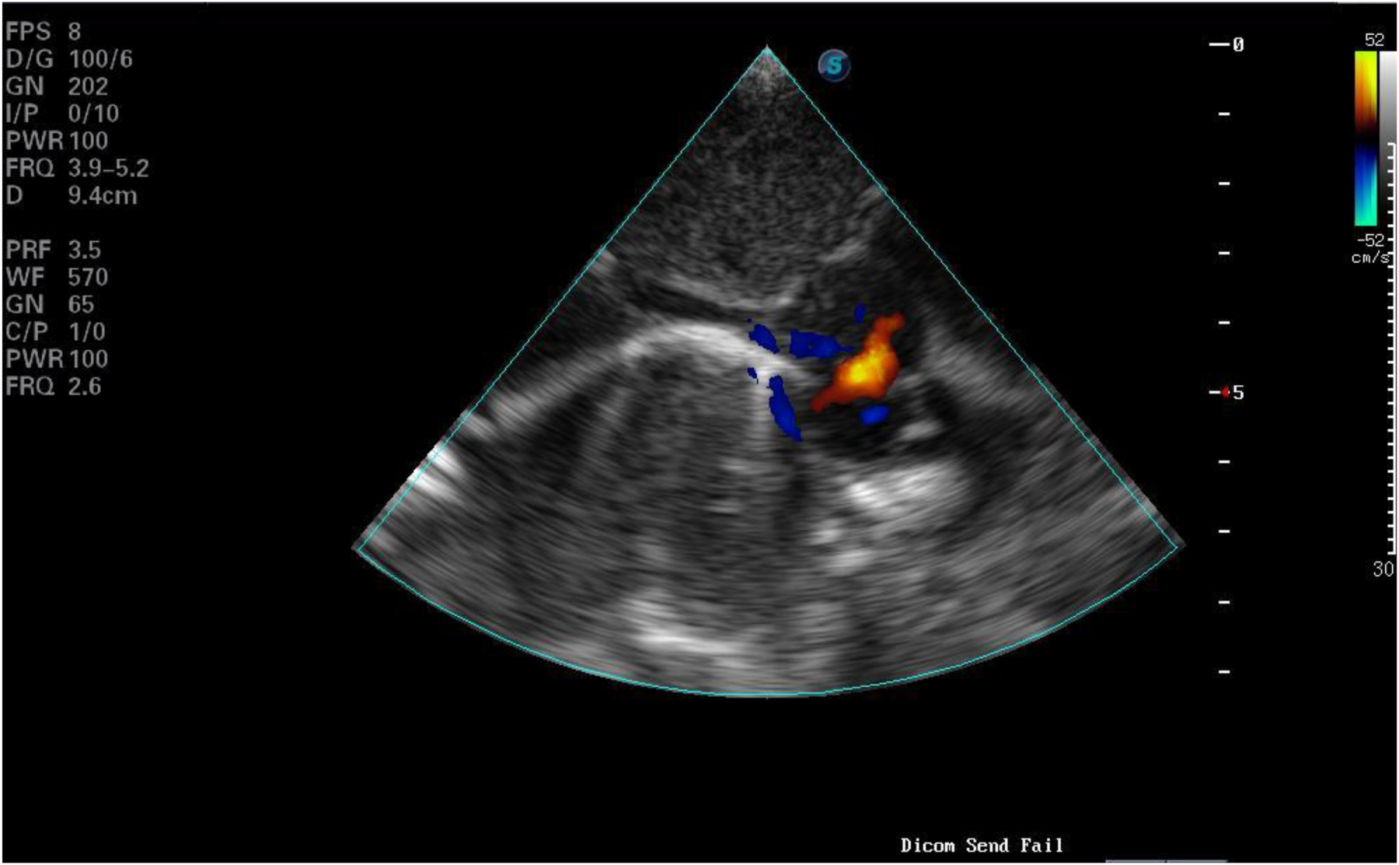
Patent ductus arteriosus.

Laboratory tests and examinations revealed metabolic acidosis with significantly elevated lactic acid levels on blood gas analysis. ECG indicated CAVB (Figure 1a). Imaging showed a 4.6 mm atrial septal defect at the foramen ovale and a 3.6 mm patent ductus arteriosus (PDA) (Figure 2, Figure 3). Left ventricular ejection fraction (LVEF) was 49%. Biomarkers including B-type natriuretic peptide (BNP), creatine kinase-MB (CKMB), and troponin I (TPI) were markedly elevated (TPI: 5.047 ng/ml, normal range <0.028 ng/ml;CKMB: 69.8 ng/ml, normal range 0–5 ng/ml; BNP: > 5,000 pg/ml, normal range <100pg/ml), consistent with heart failure.

The newborn was immediately placed in a radiation rescue table and intubated with mechanical ventilation. As early-onset sepsis could not be ruled out at this time, and the pathogen was uncertainty, laboratory tests were performed, ampicillin and meropenem were administered to combat infection, along with fasting, fluid restriction, diuretics, and inotropic agents to support the circulatory system. Atropine was used to address arrhythmia, while isoproterenol, milrinone, and dobutamine were administered to improve circulation. After these interventions, the child's temperature quickly rose to the normal range and blood pressure was maintained, but his heart rate was still low. Given that no congenital heart defects were identified and there was no family history of connective tissue disease, myocarditis could not be ruled out as the cause of the bradycardia. Consequently, hydrocortisone was administered（5 mg/kg,q8h）. The following day, intravenous human immunoglobulin (2.5 g) was given once. The child's heart rate increased from 70 to nearly 90 beats per minute. Arrhythmia was fully resolved 22 h after birth (Figures 1b,c).

With the improvement of cardiac function, other clinical manifestations also showed improvement. On the fifth day after birth, the ventilator was removed, and enteral feeding was initiated. Blood tests, including routine blood work, Coxsackie virus, enterovirus, blood culture, and lumbar puncture, were all within normal range s, ruling out sepsis, and antibiotics were discontinued. However, the child exhibited limb tremors after stimulation, and a head magnetic resonance imaging (MRI) revealed a thin corpus callosum and a right subventricular cyst with changes in the lateral ventricle. Additionally, the EEG showed moderate abnormalities, including bilateral sharp waves, sharp slow waves, and sharp-slow complexes.

**Figure 4:**
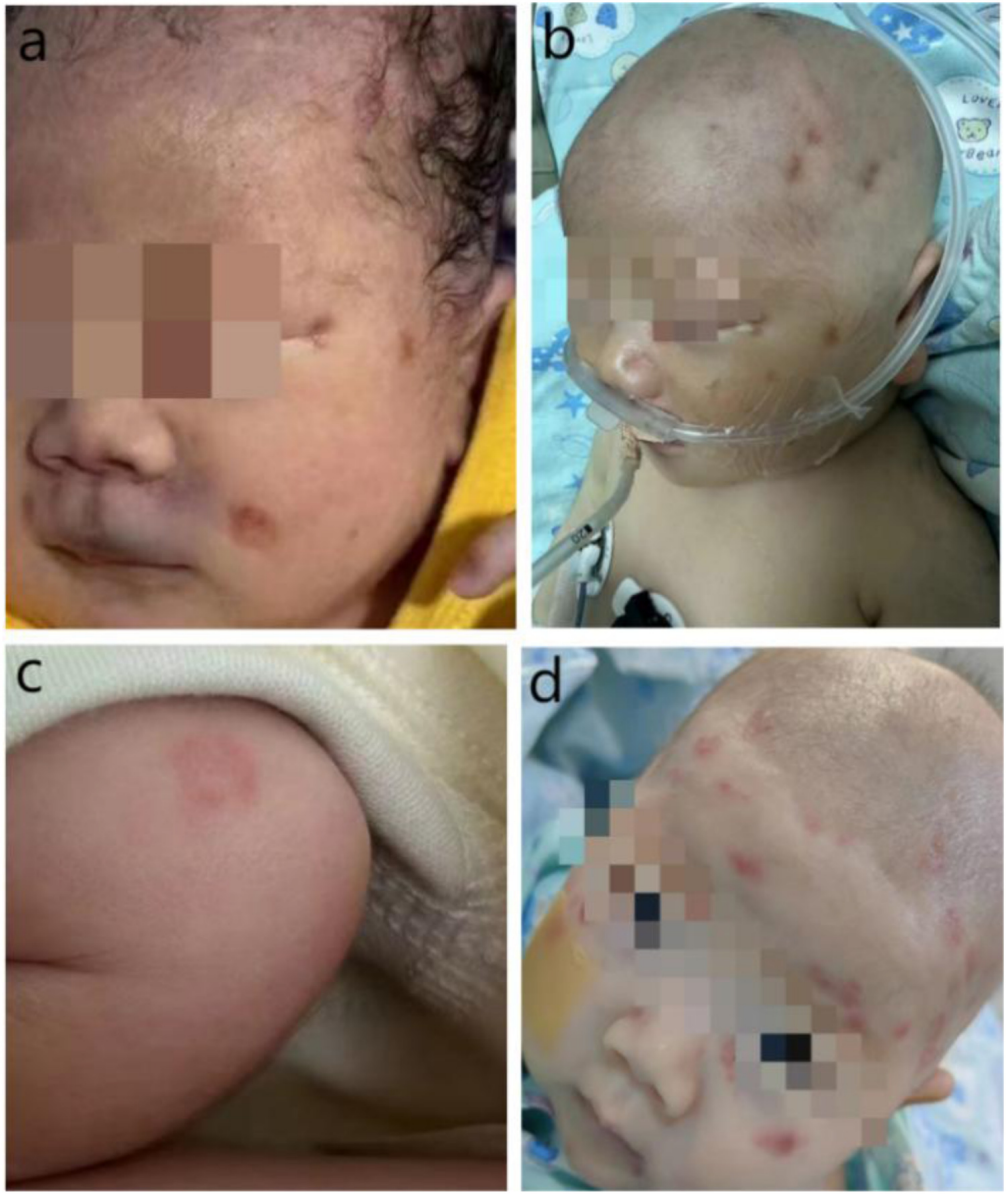
**(a)** Rash at birth. **(b)** Skin rash on the fifth day of life. **(c)** Annular erythema on the upper limb of the infant on day 38 of birth. **(d)** Erythema on the head and face of the infant on day 38 of birth.

The mother's medical history was inquired again, she had a history of multiple episodes of severe herpes both before and during pregnancy. During admission, the child presented with ecchymosis on the face. While the ecchymosis gradually subsided, the discoloration around the orbit and mouth persisted, appearing more like a rash and pigmentation. Additionally, several rash marks were noted on the scalp at the electrode sites (Figure 4b). The child was monitored for a progressive increase in HSV type 1 IgG antibody levels (Day 2: 5.87 COI; Day 5: 27.5 COI, normal range<1.1 COI), and the sequenceof HSV-1 detected by metagenomic next-generation sequencing of cerebrospinal fluid. And combine with clinical signs, including skin lesions, liver abnormalities (AST: 92 U/L, normal range 21–80 U/L;ALT: 350 U/L, normal range 8–71 U/L), myocarditis (CK-MB: 69.08 ng/ml, normal range 0–5 ng/ml), and abnormal coagulation (APTT: 51.1 s, normal range 20–39.4 s; fibrinogen: 127 mg/dl, normal range 200–400 mg/dl, prothrombin time: 16.8 s, normal range8.7–14.7 s), the child was diagnosed with HSV infection. Treatment involved acyclovir administered intravenously for 3 weeks, followed by oral acyclovir, with a total course of six months.

Following antiviral treatment, the head MRI, electroencephalogram (EEG), hearing, and retinopathy of prematurity (ROP) screening returned to normal. Before discharge, a re-examination of the cardiac ultrasound was conducted, showing an LVEF of 62%. By the 29th day after birth, the infant's body weight had increased to 2.92 kg, vital signs were stable, feeding was adequate, and there was minimal rash, leading to discharge with medication. During outpatient follow-up, however, the rash persisted and appeared to be increasing, particularly on the head, face, and upper limbs (Figures 4c,d), although HSV antibody titers were decreasing. The infant and the mother were tested for autoimmune diseases, revealing that antinuclear antibody (ANA) was weakly positive (1:100), and both were positive for anti-SSA and RO-52, leading to a diagnosis of NLE. Since the infant exhibited no systemic symptoms other than the rash, no treatment was initiated aside from recommendations to avoid ultraviolet exposure. The child was followed up regularly until the age of 7 months, by which time antiviral treatment was completed. At this stage, the rash had almost completely resolved, growth and development were normal, renal function was intact, and there were no long-term complications or neurological sequelae. However, the follow-up period remains short, and ongoing monitoring is required.

## Discussion

CAVB is predominantly etiologically linked to autoimmune disorders, infectious pathogens, and structural cardiac anomalies. When congenital CAVB is linked to maternal autoimmune disorders, antenatal administration of dexamethasone or betamethasone via the maternal route is preferred, owing to their high placental transfer efficiency. Postnatally, however, hydrocortisone is generally regarded as the safer glucocorticoid option for newborns. As the principal glucocorticoid secreted endogenously by the adrenal gland, hydrocortisone minimizes side effects associated with exogenous hormone therapy while exerting relatively mild suppression of the hypothalamic-pituitary-adrenal (HPA) axis. Additionally, its inherent mineralocorticoid activity can offset potential mineralocorticoid deficiency arising from neonatal adrenal insufficiency. In preterm infants or neonates with hypotension, hydrocortisone further contributes to blood volume maintenance and blood pressure stabilization through its mineralocorticoid-mediated effects.

Viral etiologies of neonatal atrioventricular block are primarily attributed to rubella virus, cytomegalovirus, herpes simplex virus, coxsackievirus, and parvovirus B19, among others (3). Diagnostic confirmation of viral involvement may be achieved through: Serological screening for pathogen-specific antibodies in maternal and neonatal sera; Molecular diagnostics via polymerase chain reaction (PCR) testing of blood, urine, throat swabs, or stool specimens; Histopathological assessment of placental tissue or cord blood samples for viral inclusion bodies. In the United States, the incidence of neonatal HSV infection is approximately 1 in 10,000 live births. It is classified into three main types: skin-eye-oral (35%–45%), central nervous system (CNS) infection (30%–35%), and systemic dissemination (25%–30%) (4). The primary routes of infection include exposure during delivery, ascending infection through an apparently intact fetal membrane, and postpartum transmission due to postnatal exposure to NLE (5). In this case, the child was considered to have a systemic disseminated infection likely caused by intrauterine transmission.

The clinical manifestations of neonatal disseminated HSV infection are similar to those of bacterial sepsis and lack specificity, often involving multiple organ systems. In the absence of a clear history of rash and relevant clinical symptoms, the condition is easily misdiagnosed or overlooked. Antibiotic therapy should be discontinued as soon as possible once bacterial infection has been ruled out, typically within 48–72 h (6). Common methods for detecting HSV include direct smear tests for skin lesions, conjunctiva, and oral mucosa, as well as virus isolation and culture, which are both commonly used and non-invasive (7, 8). For cerebrospinal fluid (CSF) and blood samples, quantitative fluorescence polymerase chain reaction (PCR) and serological tests each have their advantages and disadvantages. However, PCR is more sensitive and is considered the preferred test for diagnosing CNS involvement.

It is accepted that neonatal HSV infection presenting with clinical features such as skin and mucosal blisters, seizures, lethargy, respiratory distress, thrombocytopenia, coagulation abnormalities, hypothermia, sepsis-like symptoms, hepatomegaly, ascites, or significantly elevated transaminase levels requires empirical antiviral therapy. Other indicators include persistent or recurrent erythema, suppuration, or crusting at scalp electrode sites (1, 9, 10). Acyclovir remains the drug of choice for treating HSV infections. The currently recommended antiviral regimen consists of intravenous acyclovir (60 mg/kg/day, administered three times daily at 8-h intervals) for 14 days, followed by oral acyclovir (300 mg/m^2^/dose, three times daily) for a 6-month suppressive course. A multicenter trial demonstrated that continuing oral acyclovir therapy after intravenous administration significantly reduces skin recurrences and improves neurological outcomes in infants with CNS involvement (11). Recurrent HSV-related skin blisters occur in 50%–80% of neonatal patients (12). Infants experiencing three or more skin recurrences before 6 months of age are at increased risk of neurodevelopmental abnormalities at follow-up (13). Both prophylactic and therapeutic HSV vaccines remain under development (14, 15).

Additionally, the child was diagnosed with NLE, which further complicated the clinical identification and diagnosis. The diagnostic criteria for NLE include: (1) congenital heart block in neonates or the presence of anti-Ro/SSA and/or anti-La/SSB antibodies in either the neonate or the mother; (2) NLE-associated skin lesions confirmed by dermatologists and/or histopathology, with positive anti-Ro/SSA and/or anti-La/SSB antibodies in the newborn and/or the mother (16). The child met the aforementioned diagnostic criteria. However, the pathogenesis of NLE is also associated with genetic susceptibility and environmental stress (17). Even women with extremely high antibody titers can have a normal pregnancy, though they face an increased risk (18, 19).

NLE can cause systemic multiorgan damage, with the most frequently reported clinical manifestations being congenital heart block, skin lesions, and hemocytopenia. Digestive, respiratory, and neurological complications are less common. Most symptoms subside by around six months of age as maternal antibodies disappear. Cardiac involvement is the most severe complication in children with NLE, and in severe cases, CAVB or macrophage activation syndrome can be fatal. Approximately 72.9% of children with heart block are diagnosed between the 18th and 26th weeks of gestation. Heart block is more frequently observed in children from Europe (49.4%) and the Americas (35%) (20). NLE complicated by atrioventricular block (AVB) is often irreversible, and most children with complete heart block require pacemaker implantation within the first year of life (21, 22).

The management of CAVB in neonates remains controversial, necessitating individualized assessment and integrated treatment strategies based on the underlying etiology, ventricular rate, and clinical manifestations. Pacemaker implantation represents a critical life-saving intervention (23). In this case, the ventricular rate was maintained at 70–80 beats per minute, and hemodynamic stability was achieved following therapy. The arrhythmia was promptly corrected, so that pacemaker implantation was avoided. Nevertheless, congenital AVB requires long-term surveillance. Regular follow-up with electrocardiography and echocardiography is essential, alongside ongoing evaluation of growth and developmental milestones. Upon reviewing the patient's condition, it was noted that the rash recurred after discharge. Although it is difficult to entirely rule out the possibility that the AVB present at birth was caused by NLE, the rapid resolution of the AVB suggests otherwise. At present, there are studies on children with lupus erythematosus combined with herpes zoster infection, as well as reports on simple neonatal lupus (24, 25), but no cases of neonatal lupus combined with herpes simplex virus or treatment plans have been found. However, given the potential for recurrence of systemic disseminated HSV infection, which is associated with neurological damage and long-term sequelae, a multidisciplinary consultation led to the decision to continue antiviral therapy with acyclovir until the child reaches six months of age.

## Conclusions

This report identification of dual diagnoses (HSV infection and NLE) in a single neonate with CAVB and emphasizes the need for comprehensive etiological screening in complex cases. However, as a single-case report, the findings are not generalizable to neonates with CAVB of different etiologies, and and its efficacy in other cases with similar etiologies is unproven.The short-term follow-up period limits our ability to assess long-term outcomes or potential late-onset complications. Although advancements in diagnosis and treatment have significantly reduced mortality and improved long-term outcomes, further research and development are still needed. It is critical to enhance clinicians' ability to identify infants at risk of HSV infection, as well as to strengthen prenatal and maternal autoimmune antibody screening. In clinical practice, physicians should remain vigilant in cases with similar presentations, actively investigate potential causes, and thoroughly inquire into the medical history. Early diagnosis and timely treatment are essential to improving survival rates and neurological outcomes while avoiding delays in treatment.

## Data Availability

The original contributions presented in the study are included in the article/Supplementary Material, further inquiries can be directed to the corresponding author.
