# Supplementary material for: Neonatal herpes simplex virus infection combined with neonatal lupus erythematosus: a case reported
Source: Front Pediatr. 2025 Jun 12;13:1592459. doi: 10.3389/fped.2025.1592459 (PMC12198234; doi:10.3389/fped.2025.1592459)
Supplement: Supplementary file 1 [file Supplementaryfile1.pdf]

CARE Checklist of information to include when writing a case report

CARE case report guidelines

(cc) BY-NC-ND

Topic	Item	Checklist item description	Reported on Line
Title	1	The diagnosis or intervention of primary focus followed by the words "case report"	Line 1
Key Words	2	2 to 5 key words that identify diagnoses or interventions in this case report, including "case report"	Line 58
Abstract (no references)	3a	Introduction: What is unique about this case and what does it add to the scientific literature?	Line 41-44
	3b	Main symptoms and/or important clinical findings	Line 45-47
	3c	The main diagnoses, therapeutic interventions, and outcomes	Line 48-54
	3d	Conclusion—What is the main "take-away" lesson(s) from this case?	Line 55-57
Introduction	4	One or two paragraphs summarizing why this case is unique (**may include references**)	Line 79-89
Patient Information	5a	De-identified patient specific information.	Line 91-98
	5b	Primary concerns and symptoms of the patient.	Line 98-108
	5c	Medical, family, and psycho-social history including relevant genetic information	N/A
	5d	Relevant past interventions with outcomes	N/A
Clinical Findings	6	Describe significant physical examination (PE) and important clinical findings.	Line 103-116
Timeline	7	Historical and current information from this episode of care organized as a timeline	Line 91,130,132,158
Diagnostic Assessment	8a	Diagnostic testing (such as PE, laboratory testing, imaging, surveys).	Line 145-158
	8b	Diagnostic challenges (such as access to testing, financial, or cultural)	Line 135-145
	8c	Diagnosis (including other diagnoses considered)	Line 153,164
	8d	Prognosis (such as staging in oncology) where applicable	Line 166-171
Therapeutic Intervention	9a	Types of therapeutic intervention (such as pharmacologic, surgical, preventive, self-care)	Line 117-128
	9b	Administration of therapeutic intervention (such as dosage, strength, duration)	Line 153-154
	9c	Changes in therapeutic intervention (with rationale)	Line 165-166
Follow-up and Outcomes	10a	Clinician and patient-assessed outcomes (if available)	Line 158-166
	10b	Important follow-up diagnostic and other test results	N/A
	10c	Intervention adherence and tolerability (How was this assessed?)	N/A
	10d	Adverse and unanticipated events	N/A
Discussion	11a	A scientific discussion of the strengths AND limitations associated with this case report	Line 268-277
	11b	Discussion of the relevant medical literature **with references**.	Line 173-266
	11c	The scientific rationale for any conclusions (including assessment of possible causes)	N/A
	11d	The primary "take-away" lessons of this case report (without references) in a one paragraph conclusion	Line 268-281
Patient Perspective	12	The patient should share their perspective in one to two paragraphs on the treatment(s) they received	N/A
Informed Consent	13	Did the patient give informed consent? Please provide if requested	Yes ☑ No ☐
